# Assessing the causes of under-five mortality and proportion associated with pneumococcal diseases in Cameroon. *A case-finding retrospective observational study: 2006–2012*

**DOI:** 10.1371/journal.pone.0212939

**Published:** 2019-04-17

**Authors:** John Njuma Libwea, Sandrine Rachel Bebey Kingue, Nadesh Taku Ashukem, Marie Kobela, Angeline Boula, Koulla-Shiro Sinata, Paul Koki Ndombo

**Affiliations:** 1 Faculty of Social Sciences, Health Sciences Unit, Tampere University, Tampere, Finland; 2 Expanded Programme on Immunization, Yaoundé, Cameroon; 3 Mother & Child Centre (MCH), Chantal Biya Foundation, Yaoundé, Cameroon; 4 Faculty of Sciences, Department of Medical Microbiology, University of Yaoundé 1, Yaoundé, Cameroon; 5 Faculty of Sciences, Department of Microbiology, University of Buea, Buea, Cameroon; 6 Faculty of Medicine and Biomedical Sciences, University of Yaoundé 1, Yaoundé, Cameroon; 7 Ministry of Public Health, Yaoundé, Cameroon; Epicentre, CAMEROON

## Abstract

**Background:**

Vital registration data outlining causes of deaths (CoD) are important for a sustainable health system, targeted interventions and other relevant policies. There is data paucity on vital registration systems in developing countries. We assessed the leading causes and proportions of under-five deaths, and particularly those related to pneumococcal infections in Yaoundé, Cameroon, using hospital registration data.

**Methods:**

A retrospective case-finding observational study design was used to access and identify data on 817 death cases in children under-five years of age recorded in health facilities in Yaoundé, within the period January 1, 2006 and December 31, 2012. Patients’ files were randomly selected and needed information including demographic data, date of admission, clinical and laboratory diagnosis, principal and/or underlying causes of death were abstracted into structured case report forms. The International Classification of Diseases and Clinical Modifications 10^th^ revision (ICD-10-CM) codes (ICD10Data.com 2017 edition) were used to classify the different CoD, retrospectively. Ascertainment of CoD was based on medical report and estimates were done using the Kaplan-Meier procedure and descriptive statistics.

**Results:**

Of the 817 death records assessed, malaria was the leading CoD and was responsible for 17.5% of cases. Meningitis was the second largest CoD with 11.0%; followed by sepsis (10.0%), *Streptococcus pneumoniae* infections (8.3%), malnutrition (8.3%), gastro-enteritis / diarrhoea (6.2%), anaemia (6.1%) and HIV (3.5%), respectively.

**Conclusion:**

The main CoD in this population are either treatable or vaccine-preventable; a trend consistent with previous reports across developing countries. Besides, the health effects from non-communicable infections should not be neglected. Therefore, scaling-up measures to reduce causes of under-five deaths will demand sustainable efforts to enhance both treatment and disease prevention strategies, to avoid a decline in the progress towards reducing under-five deaths by 2/3 from the 1990 baseline.

## Background

Accurate civil registration systems are essential in the documentation on the distribution of CoD in children as well as in the general population. This is important in the planning of sustainable health policies and needed interventions in concordance with the millennium development goal (MDG 4) [1], with a target of reducing by two-thirds between 1990 and 2015 the under-five mortality rate [2]. We have exceeded the year 2015 deadline and globally, reports suggest the number of under-five deaths dropped from 12.7 million in 1990 to 6.3 million in 2013 [3]. However, most of the 6.3 million reported under-five deaths occurred in developing countries, with porous data on child health and mortality [4]. In these countries, reaching the MDG4 target will require an acceleration of essential, effective and affordable interventions against diarrhea, sepsis, the human immuno-deficiency virus / acquired immuno-deficiency syndrome (HIV/AIDS), malaria and pneumonia including improved nutrition and vaccines access.

The reduction of under-five mortality remains a major priority in developing countries, considering the high number of deaths resulting from preventable conditions [5]. However, to achieve the MDG4 target, reliable data are needed on under-five mortality to guide health planners and to scale up prevention and treatment strategies [5,6].

Acute Lower Respiratory Infections (ALRI) account for over 6.0% of morbidity and mortality of children worldwide, with *Streptococcus pneumoniae* reported as one of the principal causes of illness and death in children younger than five years of age [2]. About 75% of all cases occur in only 15 countries, with Sub-Saharan Africa and Southeast Asia representing the vast majority of cases [7]. Pneumococcal pneumonia is more frequent than can be confirmed by positive blood cultures and up to half of pneumonia deaths in children is attributed to pneumococcus [8]. In the absence of recent research data in the country, up to 19% of deaths in children under-five years old, have been estimated to result from pneumococcal infections in Cameroon with a total under-five mortality rate of 84 /1000 live births [9–11]. Addressing major risk factors such as malnutrition, breastfeeding and indoor air pollution are essential in prevention of pneumonia, but vaccination remains the cornerstone [12].

In this paper, we present data on hospital-based case finding of CoD in children abstracted into structured case report forms (CRF) in four health districts including the mother and child hospital (MCH) in Yaoundé, Cameroon. The MCH is one of the largest children’s hospitals in the country and it is accessible and affordable to all strata of the population. It keeps records of hospital visits, admissions, and deaths, in addition to specific clinical, laboratory and serotype data on invasive diseases including *Haemophilus influenzae* and *Streptococcus pneumoniae*. It has a capacity of 300 beds and had over 85000 admissions with 1816 reported under-five deaths between January 2006 and the end of December 2012.

The principal causes of under-five mortality in the country have not been assessed and there is no baseline data for vaccine effectiveness evaluation, as is the case with the pneumococcal conjugate vaccine introduced in July 2011. Our primary goal was to access and identify all cases of under-five deaths in the study area and define the most probable causes of under-five deaths based on available data sources. Secondarily, we estimated the proportion of deaths possibly or definitely due to pneumococcus. Moreover, it is expected the findings may be a useful baseline with information on disease pattern needed to re-scale appropriate public health targets and indicators to measure their progress and achievements in the country, with respect to the MDG4.

## Material and methods

### Study design and study site

**Fig 1:**
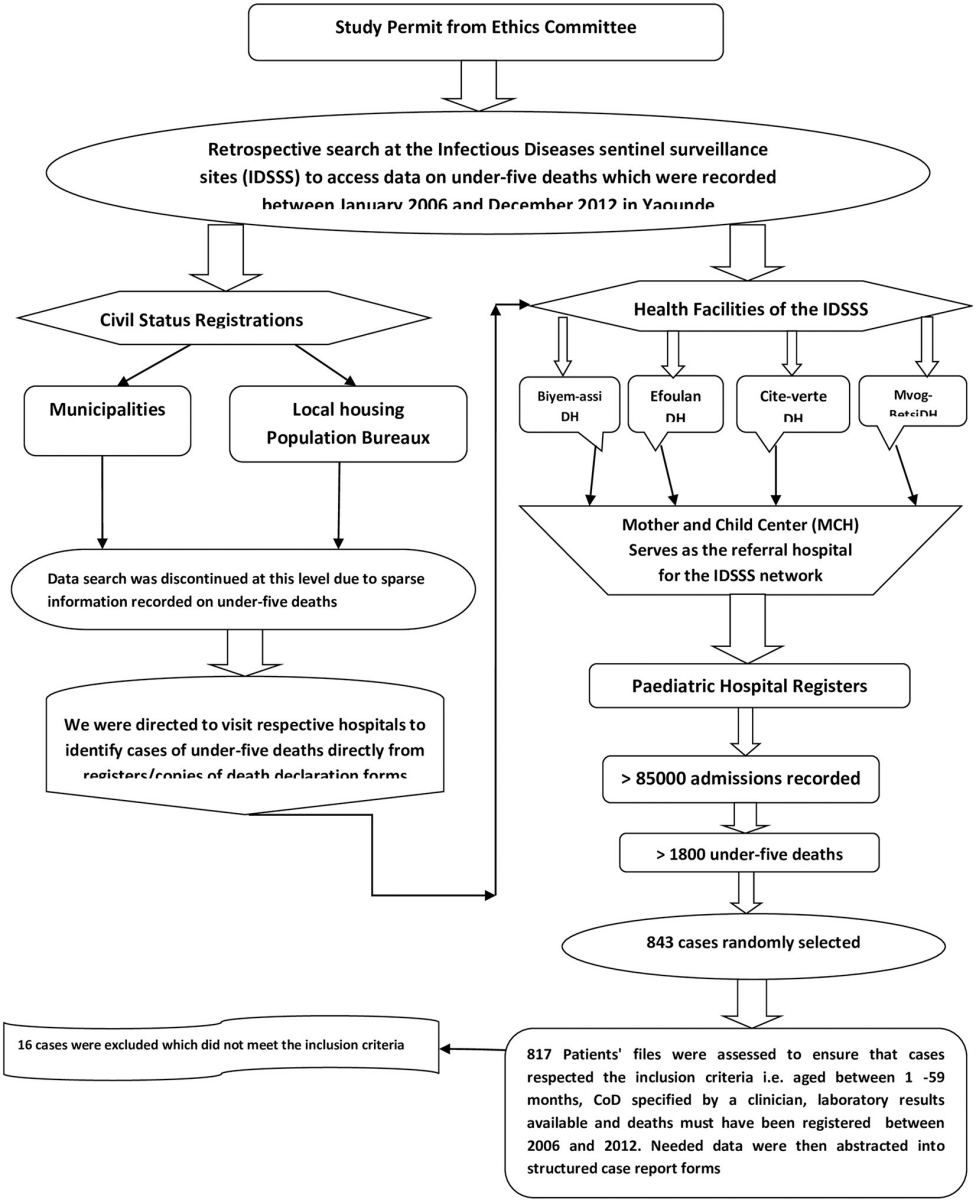
Flow chart on data identification and collection processes. N.B: For the sample size estimation, we assumed that, 18% of study population (630000) were children under-five years old; a = 0.05; power = 80%, and proportion of death cases with missing data on cause-specific death = 10%. Using the computer-based Creative Research Systems Survey software (http://www.surveysystem.com), we assumed a desired confidence level of 95% and a confidence interval of 4% units on each side; the estimated sample size for this study is 600 deaths. Therefore, a minimum of 660 cases of death in children aged 29days to 59 months was targeted as the sample size in this study. DH = District Hospital; ICD-10-CM = the International Classification of Diseases and Clinical Modifications 10th revision.

We applied a retrospective case-finding observational study design using hospital registration data from the infectious disease surveillance sites hosted at the MCH in Yaoundé, Cameroon (Fig 1). As earlier described [13], the sites involved hospitals in both an urban and rural/semi-urban zones around Yaoundé, with a population of over 3.5 million inhabitants out of which 18% are children under-five [14].

In 2013, we conducted a sample survey on over 1800 cases of death registered within the MCH, representing less than 2% of an estimated 117000 expected deaths in Yaoundé [9], among children aged under-five between 2006 and 2012. Trained study personnel randomly selected 817 of these cases and information on the socio-demographic and CoD were extracted from medical reports and keyed into structured case report forms (CRF). This facilitated the identification of the direct, intermediate and underlying causes of CoD from medical reports. Besides, it avoided duplicate or repeated counting of cases from the different hospital registers. Ascertainment of CoD was based on medical declaration and the International Classification of Diseases and Clinical Modifications 10^th^ revision (ICD-10-CM) codes were used to retrospectively classify the different CoD.

### Ethical considerations

Death registration data for the general population are held at Civil Status Registries (CSR) in municipalities and local population offices in each municipality in Cameroon, and permission for their use was obtained from the National Ethics Committee (CNE) No. 234/CNE/SE/2012, written and signed on May 2, 2012.(*Because we needed to effectively link patients’ records in registers to their respective files and to avoid duplicate entries*, *they were accessed unencoded*. *All patient records abstracted into CRF were later encoded and analysed anonymously)*. This study was approved as one of the specific objectives in a broader protocol entitled "Estimating pneumococcal disease burden and evaluating the impact of introducing the pneumococcal conjugate vaccine (PCV13) into the Expanded Programme of Immunization in Cameroon."

### Data sources for death identification

It is mandatory in the country that, in the case of death in a hospital or other medical institution or in a prison, the head of the establishment must declare the death within fifteen days [15]. The certification by medical personnel of the CoD is an essential step in the series of processes in the construction of vital registration data. In this setting, the customary practice in the registration of deaths commences with the issuance of a death declaration form (DDF) to the family of the deceased by a qualified medical staff. The family is expected to transmit the DDF to the municipality for registration and be issued a death certificate. However, this last step is hardly respected and this generates an inherent problem with the quality of data in vital registrations systems witnessed in most of resource-low settings. The recommended practice is to respect the World Health Organisation’s (WHO) criteria for medical certification; where the CoD are outlined in a sequential format beginning with the direct CoD through the intermediate causes, while the underlying CoD is registered in the lowest line of Part ׀ of the medical certificate of cause of death [10].

The data search was limited only to hospital registers because we could not find sufficient information on under-five deaths from the civil status registers (vital registration systems) in municipalities and local population offices. Generally, typical hospital setting in the country consists of independent units and wards such as the female, male, paediatric, HIV/AIDS and oncology wards. Each keeps a register of patients’ medical records (usually manual but in cities both manual and electronic formats maybe available). Our search to identify under-five hospital deaths relied principally on inpatient paediatric hospital registers, as well as on other outpatient and emergency units registers (Fig 1). This included all causes of hospital documented under-five deaths within the study period. From these, further verifications were made for any cases of death with pneumococcal aetiology.

### Case definitions

The CoD as described in hospital registers were retrospectively coded using the ICD-10-CM codes. Cause-specific mortality proportions were derived as the fraction of total deaths possibly associated to specific conditions, using hospital-based ICD-10-CM list diagnoses. Here, owing to the complexity associated with defining infections with pneumococcal aetiology, we limit our perspectives only on pneumococcal-related causes of deaths defined as follow:

A): Clinical pneumococcal infections
  1. ALRI: Diagnosis of pneumonia based on medical declaration as cause of death (i.e. any Acute Lower Respiratory Tract Infection (ALRI) or pneumonia including all diagnosis of ALRI); or
  2. Clinically severe pneumonia: Death resulting from cough or difficult breathing as admission symptoms for child 1–59 months old residing in study area,

And with either

- A respiratory rate ≥ 40/minute, or temperature >38.5°C, or refusing to feed, or vomiting and/or lower chest in-drawing

B): Laboratory confirmed infections
  - Culture-positive invasive pneumococcal disease
  - Culture-negative polymerase chain reaction (PCR) or antigen test positive invasive pneumococcal disease
C): Radiologically confirmed infections
  - Radiologically confirmed pneumonia i.e. Chest X-ray-Community Acquired Pneumonia compatible with endpoint consolidation
  - Radiologically confirmed pneumonia i.e. Chest X-ray-Community Acquired Pneumonia with any radiologic abnormality

Clinical cases of pneumococcal infections, which did not meet these criteria, were considered as non-confirmed pneumococcal diseases (unspecified).

D): Causes of deaths not related to pneumococcus
  These included other biologically related causes of under-five deaths apart from those due to pneumococcal infections e.g. Tuberculosis (TB), HIV or malaria and others.
E): Deaths due to injuries
  This consisted of under-five deaths with non-biological causes such as those resulting from injuries or accidents.

### Statistical considerations

#### Data analysis

Cases included in this analysis were children aged between 29 days and 59 months (for uniformity we have used 29 days = 1 month, since no cases were registered as 29 or 30 days old), whose deaths were recorded between 1^st^ January 2006 and 31^st^ December 2012 in the study area. Deaths occurring in the neonatal period (i.e. 28 days following birth) were excluded. The date of birth (age in months) was considered as the entry point to the study in 2006. Follow-up time was up to 2012, which was the end of the study period, or at time of death reported. The proportion and causes of deaths were estimated from data collected on the total deaths using descriptive measures and Kaplan-Meier method. Data has been analysed using the software package SPSS 25.0 version.

## Results

### Distribution and main causes of death in children under-five years old

**Fig 2:**
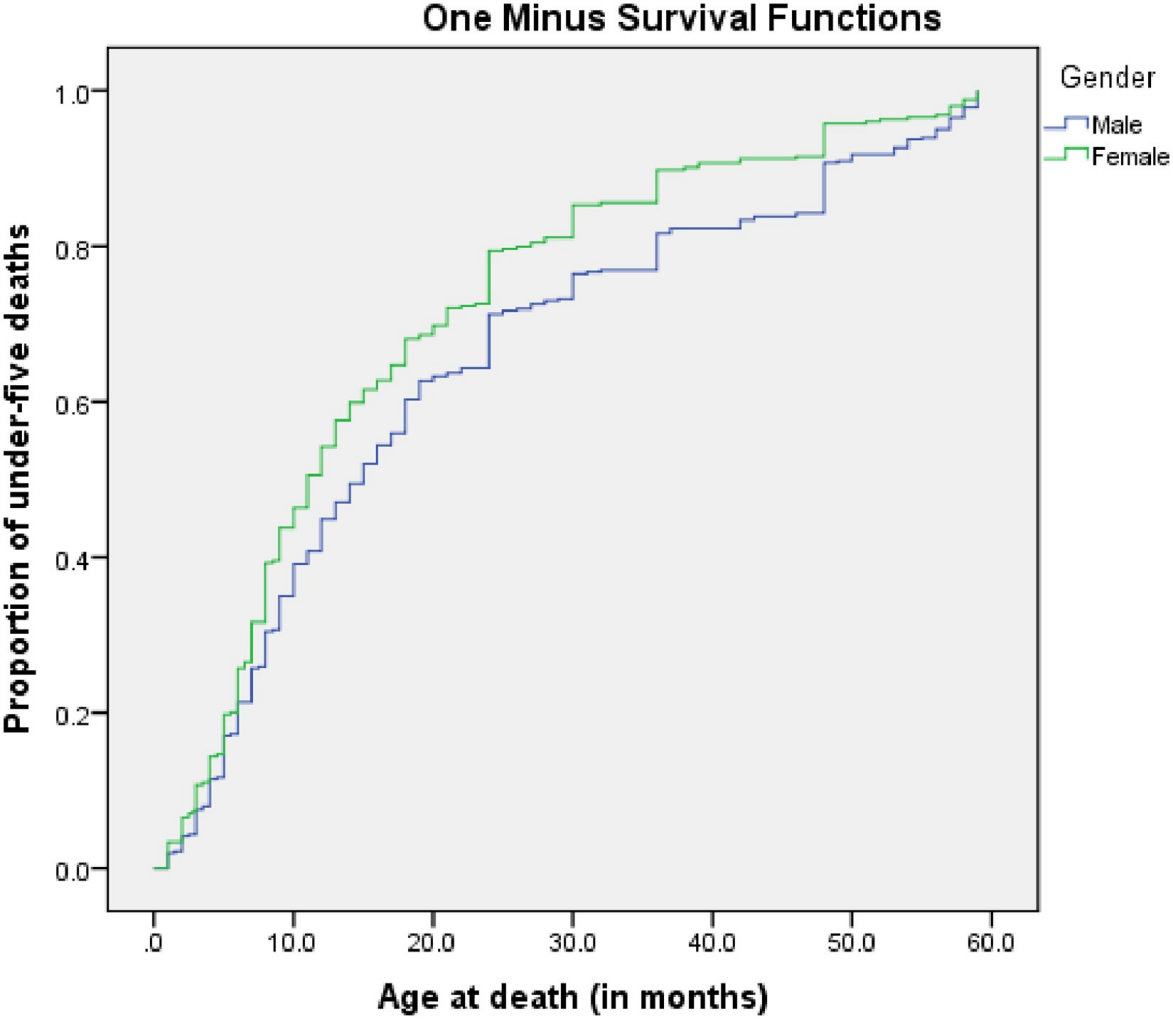
Cumulative proportion of age at death by gender among children under-five years registered at the Infectious Diseases Surveillance Sites in Yaoundé, Cameroon: 2006–2012.

A total of 817 randomly selected cases of under-five deaths from hospital registers that were recorded between January 2006 to December 2012 in Yaoundé were assessed. The median ages at death were 11months for males and 15 months for females (Fig 2).Table 1 shows the age groups and distribution of the principal causes of death. The most vulnerable were those in the age group 1–11 months old, harbouring 45% of all-cause mortality. Malaria (17.5%) was the leading CoD followed by meningitis (11.0%), sepsis (10.0%), and pneumococcal diseases (8.3%).

**Table 1:** Distribution of the main CoD in children 1–59 months old in Yaoundé, 2006–2012 (N = 817).

CoD	Age groups					
	1–11	12–23	24–35	36–47	46–49	All
	(N = 368)	(N = 187)	(N = 104)	(N = 55)	(N = 103)	(N = 817)
	%	%	%	%	%	% (95%CI)
Malaria	11.7	22.5	21.2	32.7	17.5	17.5 (15.0–20.3)
Meningitis	11.7	10.2	16.3	5.5	7.8	11.0 (09.0–13.4)
Sepsis	13.0	11.2	6.7	0.0	5.8	10.0 (08.1–12.3)
Pneumonia	9.8	7.0	8.7	3.6	3.9	8.3 (06.5–10.4)
Malnutrition	7.6	12.8	7.7	0.0	3.9	8.3 (06.5–10.4)
Diarrhoea/gastro-enteritis	8.7	5.3	4.8	1.8	2.9	6.2 (04.7–08.1)
Others	37.5	31.0	34.6	56.4	58.3	38.6 (35.2–42.0)

CoD = Causes of death; N = number; % = percentage; CI = Confidence Interval

### Causes of death registered with corresponding ICD-10-CM codes

Table 2 shows a summary distribution of the 817 cases of under-five deaths classified by ICD-10-CM coding. Communicable infections resulting from infectious and parasitic diseases, respiratory infections, nutritional deficiencies and perinatal conditions were accountable for most deaths (71.5%). Non-communicable infections including those from malignant disorders, cardiovascular disorders and congenital abnormalities contributed for some 11.6%, anaemia contributed to 6.2% of deaths and injuries were responsible for 0.2%.

**Table 2:** List of ICD-10-CM codes collected for this study and percent of diagnostic CoD in children 1–59 months old in Yaoundé, 2006–2012.

No.	ICD-10-CM* code	Clinical diagnosis	%
**1.**	B54	Unspecified malaria	17.5
**2.**	G00.1	Pneumococcal meningitis	11.0
**3.**	A41.9 / B96.29	Sepsis	10.0
**4.**	B95.3 / J13	*Streptococcus pneumoniae* as the cause of diseases classified elsewhere	8.3
**5.**	C83.7**/**C95/D17.9/R59.1	Malignancy / Tumours/ Generalized lymph nodes	8.2
**6**	E46	Unspecified protein-calorie malnutrition	8.3
**7.**	K52.1/K52.89/R19.7	Gastro-enteritis / Diarrhoea	6.2
**8.**	D61.2	Aplastic anaemia due to other external agents	6.1
**9.**	P96.89	Other specified conditions originating in the perinatal period	4.4
**10.**	B20	Human immunodeficiency virus (HIV) disease	3.5
**11.**	A15.5	Tuberculosis of larynx, trachea and bronchus	2.3
**12.**	I25.5	Ischemic heart diseases	2.3
**13**	D57.1	Sickle-cell disease without crisis	1.1
**14.**	T14.90	Injury	0.2
**15**	R99/A35/B05.9/K75.9	Ill-defined and unknown cause of mortality and others	10.0

*ICD codes specific for diseases and compatible with clinical diagnosis as primary cause of death used in the study;

No. = Serial number based on leading cause of death; S = Streptococcus; % = percent

## Comparison of leading CoD in our study with estimates from Sub-Saharan Africa (SSA)

**Fig 3:**
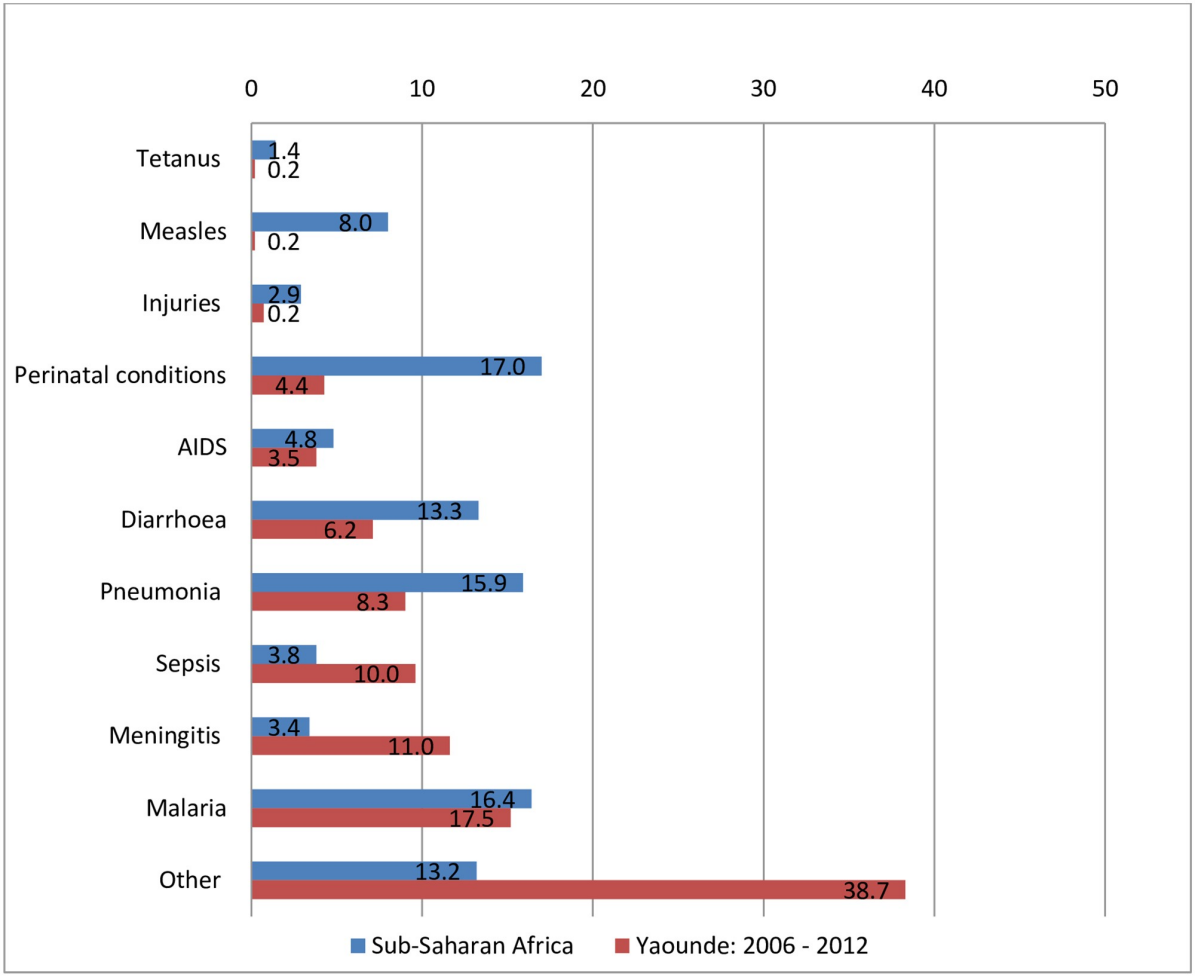
Frequent causes of deaths in our study with comparative percentages for the Sub-Saharan Africa region.

In Fig 3, are presented percentages of cause-specific deaths of children age 1–59 months obtained in our study compared to estimates from the SSA region [3]. Meningitis and sepsis were contributing more cause-specific deaths in our study population compared to the rest of the sub-region. The statistics were similar for malaria and HIV/AIDS. Some positives are observed with a decline in CoD resulting from tetanus, measles and perinatal conditions in our study. Deaths attributed to other and ill-defined causes were as much as threefold higher in our study compared to that of the entire SSA region [3].

### Clinical and laboratory diagnosed pneumococcal disease associated cause of death

Table 3 presents the distribution of clinically diagnosed and laboratory confirmed pneumococcal disease associated CoD. Overall, more than 55% of cases (n = 158) diagnosed with clinically severe pneumonia had been coughing, had a respiratory rate ≥ 40/minute and a body temperature of over 38°C. Seventeen percent of these were confirmed as culture-positive invasive pneumococcal disease, and 73.4% were confirmed as antigen test-positive. Only 1.3% of these had a radiological confirmation.

**Table 3:** Distribution of pneumococcal disease associated deaths by study case definition in children 1–59 months old in Yaoundé, between January 2006 to December 2012 (N = 158).

Diagnostic algorithm	YES		NO		UNKNOWN/ MISSING	
	n	%	n	%	n	%
**Clinically severe pneumonia**						
Cough	89	56.3	16	10.1	53	33.5
Respiratory rate ≥ 40/minutes	119	75.3	12	7.6	27	17.1
Temperature ˃ 38°C	114	72.2	24	15.2	20	12.7
Refusing to feed	62	39.2	25	15.8	71	44.9
Vomiting /lower chest in-drawing	57	36.1	6	3.8	95	60.1
**Laboratory confirmed pneumococcal disease**						
Culture positive IPD*	27	17.1	4	2.5	127	80.4
Culture negative PCR^♠^/ antigen test positive IPD	116	73.4	10	6.3	32	20.3
**Radiologically confirmed infection**						
X-ray CAP^♣^ with endpoint consolidation	2	1.3	1	1.6	155	98.1
X-ray CAP without endpoint consolidation	0	0	0	0	0	0

*Invasive pneumococcal disease;

^♠^ = polymerase chain reaction;

^♣^ = Community acquired pneumonia; N (n) = number; % = percentage.

Endpoint consolidation refers to the presence of alveolar or pleural effusion on chest X-ray

## Discussion

This study aimed to determine the leading CoD in children aged 1–59 months in the MCH and health facilities within the infectious disease surveillance network in Yaoundé, Cameroon, using hospital-based registers. Vital statistics regarding the CoD among children based on death certificates are porous in resource-limited settings like Cameroon, which remain amongst the highest contributors to the number of under-five reported deaths [16]. In this study, we have assessed only about 2% of deaths of an estimated 117000 deaths expected to have occurred within the study period in Yaoundé [9], justified by our intention to capture CoD with clinical and laboratory-culture specific data to depict deaths from pneumococcal related aetiology. Thus, we could not ascertain that these deaths were representative of all deaths within this age group at that period of time, but we remain optimistic they mirrored the major causes of morbidity and mortality in this community since they were medically declared. Based on the data we assessed, infectious diseases and malnutrition were the leading CoD in children aged 1–59 months within the study period in this population. Additionally, 71.5% of the deaths occurred within the first 24 months of life.

However, our findings are in agreement with previous reports on the trends in the geographical distribution of CoD in the under-fives globally [16,17], although these studies had explored various methodologies than what we have used. This is partly due to the absence of a standardized universal algorithm to measure CoD.

In a related study on the causes and circumstances of death in a district hospital in Kolofata, Northern Cameroon between 1993 to 2009; malaria (15.9%), infectious diseases including ALRI (15.1%) and diarrhoeal diseases (13.3%) were the leading CoD in children before the age of 5 years [18], a similar trend to that obtained in our study. The Kolofata study included 1281 inpatient deaths of which 46.9% were deaths before the age of five years. Although there was a decline in the under-five mortality rate in the country from 144/1000 live births reported in 2004 to 122/1000 live births in 2011 [9], country-specific data on the major causes of death are scarce [18]. Vital statistics with notifications on CoD in children are not maintained in Cameroon, despite legislative provisions. However, the 2011 Cameroon Demographic Health Survey (CDHS) documented important information on symptoms and other characteristics leading to the death for children aged under-five years old nationwide [9]. Based on the CDHS data, ALRI, particularly those with pneumococci aetiology had 7% prevalence in Yaoundé, similar to the 8.3% we have obtained but differ with the 16% reported for the SSA region where diarrhoea (21%), malaria (30%), malnutrition (33%) and anaemia (60%) were the predominant CoD in children aged under-five years (Fig 3).

In the CDHS questionnaires, to ascertain pneumococcal-related causes of deaths caretakers were asked the following; *if the child was coughing in the last two weeks prior to death and if yes*, *was the cough accompanied with difficult and fast breathing prior to death*? This reflects the present case definition on pneumococcal related deaths we have adopted in this study, in addition to laboratory confirmation (Table 3).

In both the CDHS and Kolofata studies, malaria and diarrhoea were the primordial causes of death in children of the age groups 1–11 months and 12–23 months old than were deaths from pneumococcal diseases. In our study, 68% of deaths also occurred within similar age groups i.e. the first 24 months of life. This, according to the CDHS 2011 report may be explained by the period when the child (at ≥ 6 months) begins to receive other nutritional components than breast milk. The latter age group also corresponds to the age when the children begin to exploit their environment, and as such exposes them to contamination with pathogens. Another contributing factor on the prevalence of diarrhoea is that of the non-availability of potable water source. Based on the CDHS 2011 report, 19% of children from homes with a potable water source were exposed to diarrhoea infection as compared 24% in those from homes without potable water within this population.

Further, related studies conducted in other parts of Africa demonstrated that communicable infections including malaria, diarrhoea, respiratory tract infections, HIV/AIDS, TB and fever of unspecified origin accounted for over 50% of death in children before their first birthdays [19–23]. More so, a study on the global and regional CoD reported in 2009 that an estimated one in two deaths in children from SSA is attributed to communicable diseases as the primary cause [24].

In our study, malaria due to *Plasmodium falciparum* was the most important contributor to under-five deaths. As many as 18 of a hundred children from this population died from malaria. This is surprising when we consider the huge investments on malaria control programs in the country, just as in most of SSA [25]. Reducing the proportion of deaths due to malaria relies partly on the preventive and treatment regimens currently in use [25]. With support from the Global Fund for Health, the government of Cameroon besides the distribution of long-lasting insecticide-treated mosquitoes bed nets to expectant mothers, is implementing the use of artemisinin-based combination therapy (ACT) as first-line malaria treatment in replacement of chloroquine [9,18]. We have limited understanding on this disproportional trend between malaria as the primordial CoD against on-going treatment and preventive measures being implemented for two decades rolling. However, there is a hypothesis that increasing resistance to antimalarial therapies and their improper use are contributing factors [9,26].

Bacterial meningitis (pneumococcal meningitis), was the second highest CoD, and occurred mostly in the age groups 1–11 and 24–35 months. The implication of the pneumococcus as the predominant organism causing bacterial meningitis had earlier been reported [27–30]. We were not surprised with this finding since the country is situated along the “African meningitis belt”, and shares a long and porous border with Nigeria, where meningococcal disease incidence is high [31]. With the advent of the monovalent serogroup A meningococcal conjugate vaccine (MenAfriVac) in the country since 2009, other pathogens such as *Streptococcus pneumoniae* and *Haemophilus influenzae* are reported to cause more infections than was previously thought [9,31].

Septicaemia with ICD-10-CM codes *A41*.*9* and *B96*.*29* was responsible for 10.0% of deaths and is seen as another major contributor to under-five deaths in this community. Most of the sepsis resulted from bacterial infections with different aetiologies acquired during the neonatal period; subsequently leading to the hospitalisation and death of the children in their early infancy. This finding aligns with previous reports that, in a malaria endemic region like Cameroon, affected children have an increased risk to die from septic shock resulting secondarily from severe anaemia and/or bacteraemia [32,33]. More so, sepsis is described as a cascade of diseases involving a systemic inflammatory response syndrome (SIRS) in the midst of infection, intensifying in septic shock to cardiovascular and organ system dysfunction [34]. Some authors have attributed this systemic organ dysfunction as the last inflammatory stage for a majority of infectious disease-related paediatric deaths globally [34–36]. This phenomenon is consistent with our findings in which a myriad of infections resulting from HIV/AIDS (3.5%), *Streptococcus pneumoniae* (8.3%), malignancies (8.2%), ischemic heart diseases (2.3%), conditions from perinatal period (4.4%) and aplastic anaemia due to other external agents (6.1%) could be important underlying contributors to under-five deaths, although they were recorded as individual CoD (Table 2). We also recognize that multiple diagnostic CoD are a possibility, especially in a remote setting where patients present with concomitant infections as previously reported [37]. We have reported just a single CoD in this study based on the primary CoD with respect to medical declaration. However, to use a single CoD as we have done turned not to underestimate the contributions of the individual causes to the overall under-five mortality rate which witnessed a decline from 146/1000 live births in 2001 to 122/100 live births in 2011 [9].

Pneumonia due to *Streptococcus pneumoniae* (8.3%) was the fourth most important CoD, with most of the deaths occurring in children aged between 1–11 months. This observed aged pattern of pneumococcal related deaths highlights the importance of the pneumococcus as a serious health challenge to children in SSA including Cameroon [1,38].

Malnutrition related deaths which accounted for 8.3% overall CoD in our study, are a baseline to causes of child death in Africa [39]. In our data, chronic malnutrition usually protein-calorie malnutrition (with ICD-10-CM code: *E46*), is translated by a small height-for-age index which corresponds to growth retardation in children [9]. There are some variations in the prevalence of malnutrition by age, which is highest in the age group 12–24 months old (Table 1). We lack direct answers on why this form of malnutrition was predominant since our study was not designed to address risk factors for malnutrition. However, according to the CDHS 2011 survey this may be because of inadequate food intake and / or attributed to recurrent or relatively long periods of infectious diseases [9]. More so, for children with such growth retardation it is evidenced that after the age of two years, there is a limited chance to ameliorate their growth with any nutritional intervention [40]. The height-for-age index is a measure of the long-term effect of malnutrition and is a useful indicator of the environmental, developmental and socio-economic level of a given population [40]. Thus, ameliorating the challenges of malnutrition should lead to a reduction in the number of under-five deaths in this population, and subsequently an improvement in their living standards.

Diarrhoea associated CoD was another predominant contributor to under-five mortality in the study area with 6.2% of all deaths. Majority of the deaths occurred between the age group 1–11 months. This is lower than the 13.3% prevalence reported in the Kolofata study in Northern Cameroon [18]. In a related study in Haiti, diarrhoea was found to be responsible for up to 60% of deaths among those in the age bracket 1–11 months old [4]. Hospital-based diarrhoea associated CoD is reported to vary in SSA from between 2% to about 40%, with most of the deaths occurring before the children reach their first birthdays [41]. However, the 6.2% prevalence obtained in our study shows a decline in diarrhoeal associated CoD when compared with the 13% aggregated data obtained from SSA region (Fig 3).

In our data, 8.2%, 6.1%, 4.4% and 2.3% of deaths were respectively, due to malignancies/tumours/generalized lymph nodes, anaemia, and diseases originating from perinatal conditions and tuberculosis (Table 2). The contributions of non-communicable diseases including malignancies/tumours and/or generalized lymph nodes to under-five deaths in the Yaoundé study area should not be undermined. Previous studies on causes of under-five mortality in SSA have scarcely highlighted the role of malignancies and related CoD, as there is too much focus on communicable infections [1,18,34,35,38].

### Strength and limitation

There are some shortcomings in this study. First, we have only considered a single CoD as ascertained by a clinician, despite the understanding that in many resource-low settings a cascade of infections may be involved in a specific CoD [37]. Our main difficulty was the inability to ascertain the exact order in sequence of events leading to a specific CoD. However, the advantage here is that clinicians with long years of experience in tropical and paediatric medicines clinically validated the CoD. In addition, the methodology we used did not require subjective assessments from caretakers or open-ended algorithms in the determination of the primary CoD. Hence, compromising issues on measurement bias as it is common with the verbal autopsy technique. Further, we could not incorporate the verbal autopsy technique that should have addressed some of the deaths, which occurred outside the hospital settings, even if this too is plagued with inherent limitations. More so, our methodology may be useful to monitor possible changes in mortality trends considering that access to the hospitals were opened to everyone in the communities.

### Recommendations and conclusion

The analyses and estimates we have presented are to support a better understanding on country-specific CoD in children under-five years old in a resource-limited setting, where vital registration system is weak to provide valid estimates on CoD distribution among the populations. The current estimates on the CoD we have reported are consistent with diagnoses based on medical declaration as ascertained by clinicians. Such estimates and information are needed for the planning of child health priorities, possible intervention and subsequently, a baseline for evaluation studies. In understanding the aetiology of the main primary causes of under-five mortality in our study, we from this basis suggest possible steps needed to reach the MDG4 objectives (Box 1).

**Box 1. Recommendations on some essential points on reaching the MDG4 goal in Cameroon**

- Increase in health funding/budget to facilitate the amelioration of health infrastructure and workforce through an enhanced community-based framework
- Establishment of a sustainable multi-sectoral approach to integrate child health, nutrition and social welfare policies and programmes for poverty reduction, agricultural improvements, food security and safety; upgrading of water, hygiene and sanitation systems.
- The experience of Ethiopia [42] that was used as a case study to the 2015 MDG countdown could be successfully applied to similar settings in Cameroon.
- Maternal factors such as education, dietary intake and nutritional status are strong determinants to child health outcomes, which should be carefully considered.
- There is an eminent risk of an increase of the ‘triple burden of disease’ i.e. the concomitant high burden of infectious diseases, malnutrition and non-infectious diseases. Appropriate cost-effective measures need to be established to counter this trend.
- Health administrators need to recognize what worked well and use lessons from the MDGs to address challenges in future health interventions, and outline roadmaps to achieve specific indicators
- Necessity to re-orientate targeted strategies, interventions and programmes by critically analysing evidence on the epidemiology of causes of death.
- Restructuring of health investment and financing mechanisms as an essential part of health intervention.
- Educating the public to follow-up with the required rules so that proper registry can be built for future generations
- Political commitment, such as the enforcement on the legislation of civil status registration systems and human resource training and mobilisation at the community level.

Our findings demonstrate that the main CoD among the inpatient deaths assessed in these hospitals are either treatable or vaccine-preventable. This trend is consistent with previous reports across developing countries. Therefore, scaling-up measures to reduce causes of under-five deaths will demand sustainable efforts to enhance both treatment and disease prevention strategies.

## Supporting information

S1 Dataset (SAV)
